# Propranolol Suppresses Proliferation and Migration of HUVECs through Regulation of the miR-206/VEGFA Axis

**DOI:** 10.1155/2021/7629176

**Published:** 2021-10-16

**Authors:** Ting Zhang, Yingying Qian, Chunyu Yuan, Yafen Wu, Hua Qian, Hui Lu, Cui Hu, Wei Li

**Affiliations:** Department of Dermatology, Children's Hospital of Soochow University, Suzhou, Jiangsu Province 215025, China

## Abstract

Propranolol has been used in the first-line therapy of infantile hemangioma (IH) for a number of years; however, the mechanisms through which propranolol regulates IH are not yet fully understood. In the present study, microRNA (miRNA/miR) sequencing analysis was performed to identify differentially expressed miRNAs in human umbilical vascular endothelial cells (HUVECs) treated with propranolol. Cell viability and apoptosis were detected using CCK-8 assay and flow cytometry, respectively. Cell migration was assessed using wound healing, Transwell, and tube formation assays. Methylation-specific PCR was then used to investigate the promoter methylation status. The levels of oxidative stress indicators, including superoxide dismutase, glutathione, and malondialdehyde were also detected. Finally, cell cycle analysis was performed using flow cytometry and western blotting. It was observed that propranolol induced the upregulation of miR-206 in HUVECs, which was caused by demethylation of the miR-206 promoter. Moreover, propranolol significantly inhibited the proliferation of HUVECs by inducing apoptosis, while these phenomena were reversed by miR-206 antagomir. VEGFA was found to be a target gene of miR-206. In addition, propranolol notably inhibited the migration and induced G1 arrest of the HUVECs, whereas these results were eliminated by miR-206 antagomir. Collectively, the findings of the present study demonstrated that propranolol may inhibit the proliferation and migration in HUVECs via modulating the miR-206/VEGFA axis. These findings suggest a novel mechanism through which propranolol suppresses the progression of IH.

## 1. Introduction

Infantile hemangioma (IH) is the most frequent vascular tumor occurring in infancy, and it affects ~5-10% of mature neonates [1]. IH begins with the hyperplasia of endothelial cells, which is followed by a period of extensive proliferation and finally the involution phase [2]. In general, >20% of the IHs are associated with severe complications, such as ulcerations, visual impairment, airway obstruction, and congestive heart failure [3]. Therefore, accurate diagnostic tools and effective therapies are urgently required. Since the discovery that propranolol can inhibit the growth of severe hemangiomas in 2008 [4], propranolol has been widely used as a first-line therapy [5]. Propranolol is a *β*-adrenergic receptor antagonist, which can reduce heart rate and cardiac contractility; thus, it is widely used in the treatment of cardiac-cerebral vascular diseases [6–8]. However, the poor responsiveness and recurrence in some patients require the identification of novel drugs for future therapy [9, 10].

MicroRNAs (miRNAs/miRs) are classified as single-strand noncoding RNAs expressed widely during physiological or pathological processes [11]. In recent years, the role of miRNAs in cancers has been extensively investigated. They are well known to play a role in cancer cell proliferation [12], migration [13], apoptosis [14], and in other cellular processes. Meanwhile, miRNAs are known to mediate the progression of IH. For instance, miR-33a-5p could inhibit the tumorigenesis of IH via targeting HIF1*α* [15]; miR-196b-5p could facilitate intercellular interaction in IH [16]. However, the role of miRNAs in IH warrants further investigation. In the present study, the differentially expressed miRNAs in propranolol-treated human umbilical vascular endothelial cells (HUVECs) were analyzed. Furthermore, the association between propranolol and the differentially expressed miRNAs in HUVECs was investigated. The findings of the present study provide a more theoretical foundation for the propranolol treatment of IH. It is hoped that these findings may also aid in the development of novel drugs for IH therapy.

## 2. Materials and Methods

### 2.1. Cell Culture and Transfection

HUVECs were obtained from the American Type Culture Collection (ATCC) and cultured in EGM™ Endothelial Cell Growth Medium (Lonza Group, Ltd.) in a humidified incubator at 37°C with 5% CO_2_. The cells were then incubated with 0, 20, 40, 60, 80, or 100 *μ*M propranolol (Sigma-Aldrich; Merck KGaA) for 48 h, followed by analyses using various assays. miR-206 agomir, miR-206 antagomir, and negative control (Guangzhou RiboBio Co., Ltd.) were transfected into the HUVECs using Lipofectamine 2000® (Thermo Fisher Scientific, Inc.).

### 2.2. Cell Viability Assay

Cell viability was determined using Cell Counting Kit-8 (CCK-8) assay (Nanjing KeyGen Biotech Co., Ltd.). The cells were plated in a 96-well plate and treated with the reagents for 48 h; CCK-8 reagent was then added to the cells for a further 2 h at 37°C. The absorbance was then read at 450 nm using a Victor3™ microplate reader (PerkinElmer, Inc.).

### 2.3. RNA Sequencing and Analysis of Differentially Expressed miRNAs

The HUVECs were treated with or without propranolol and harvested for RNA extraction using TRIzol® reagent (Invitrogen; Thermo Fisher Scientific, Inc.) following the manufacturer's guidelines. The concentration of RNA was then quantified using a Nanodrop 2000 spectrophotometer (Thermo Fisher Scientific, Inc.). RNA library data were generated from the Illumina Hiseq platform (Illumina, Inc.). DESeq2 was used for the analysis of differentially expressed miRNAs [17]. The selection criteria for the upregulated and downregulated miRNAs were a *P* value < 0.05 and fold change > 1.2, and a *P* value < 0.05 and fold change < 0.083, respectively.

### 2.4. KEGG and GO Analysis for the Protein Targets of Differentially Expressed miRNAs

The protein targets of differentially expressed miRNAs were analyzed using miRTarBase. ClusterProfiler software was used for Gene Ontology (GO) and Kyoto Encyclopedia of Genes and Genomes (KEGG) analysis. The GO database (http://www.geneontology.org/) was used to annotate the functions of protein targets of differentially expressed miRNAs. The KEGG database (https://www.genome.jp/kegg/) was used to determine the potential pathways of the protein targets of differentially expressed miRNAs.

### 2.5. Reverse Transcription-Quantitative PCR (RT-qPCR)

RNA extraction from HUVECs was performed using TRIpure RNA Extraction Reagent (ELK Biotechnology, Co., Ltd.). First, RNA was reverse transcribed into cDNA using the EntiLink cDNA Synthesis kit (ELK Biotechnology, Co., Ltd.). The mRNA level was then determined using SYBR-Green (ELK Biotechnology, Co., Ltd.) on a StepOne™ PCR System (Thermo Fisher Scientific, Inc.). The specific primers used were as follows: miR-206 forward, 5′-TGGAATGTAAGGAAGTGTGTGG-3′ and reverse, 5′-CTCAACTGGTGTCGTGGAGTC-3′; VEGFA forward, 5′-GAACTTTCTGCTGTCTTGGGTG-3′ and reverse, 5′-GGCAGTAGCTGCGCTGATAG-3′. U19 and *β*-actin were used as the internal controls for miR-206 and VEGFA, respectively. The relative mRNA levels were quantified using the 2^-*ΔΔ*Cq^ method [18].

### 2.6. Methylation-Specific PCR

HUVECs were treated with 80 *μ*M propranolol for 48 h and then harvested for DNA extraction using the Genomic DNA Extraction kit (ELK Biotechnology, Co., Ltd.). Subsequently, DNA was subjected to bisulfite modification following the manufacturer's protocols (Methylation-Gold kit; Zymo Research Corp.). The primers for methylated (M) and unmethylated (U) PCR were synthesized by Sangon Biotech, Co., Ltd. and were as follows: M forward, 5′-TTGTATAAGAATAAGTTAGGGAAACG-3′ and reverse, 5′-CCCAAACAAAAAACTCTTAACG-3′; and U forward, 5′-GTTGTATAAGAATAAGTTAGGGAAATG-3′ and reverse, 5′-TACCCAAACAAAAAACTCTTAACA-3′. Finally, 2% agarose gel was used to separate the PCR products.

### 2.7. Flow Cytometry

HUVECs were collected and resuspended in PBS supplemented with 0.5% FBS and 2 mM EDTA. After washing with PBS, the cells were stained using the Annexin V-FITC/PI double staining kit (Nanjing KeyGen Biotech. Co. Ltd.). PI/RNase staining buffer (BD Biosciences) was used for cell cycle analysis. All steps were performed according to the manufacturer's instructions. Detection was performed using a flow cytometer (BD Biosciences) within 1 h, and the data were analyzed using FlowJo 7.6 software (FlowJo LLC).

### 2.8. Wound Healing Assay

HUVECs were seeded in a 12-well plate until they reached a confluency of 80%. A scratch was then made in a straight line in the cell layer using a 100 *μ*l pipette tip. The cells were then washed twice with PBS to remove the suspended cells and cultured in complete medium. The same scratch area was photographed at 0 h and 48 h under a microscope (Olympus Corporation). Finally, the wound healing rate was calculated according to the change in the wound area [19].

### 2.9. Transwell Assay

The Transwell migration assay was performed using 24-well Transwell chambers with an 8 *μ*m pore size (Corning, Inc.). First, 200 *μ*l HUVECs in serum-free medium was added to the upper chamber of the well, and 600 *μ*l complete medium was added to the lower chamber. The unmigrated cells on the upper surface were then removed using a cotton swab, and the migrated cells on the lower surface were stained with 0.2% crystal violet following 24 h of incubation. The images were captured under a light microscope (Olympus Corporation).

### 2.10. Tube Formation Assay

Matrigel™ (BD Biosciences) was applied to assess the tube formation ability of the HUVECs. The 6-well plates were precoated with Matrigel at 37°C for 1 h. The HUVECs were then seeded into these wells and incubated for 24 h. The images of tube formation were captured under a microscope (Olympus Corporation). Branching points and capillary lengths from three random fields were analyzed using WimTube software (Wimasis).

### 2.11. Dual-Luciferase Reporter Assay

The potential association between miR-206 and VEGFA was predicted using bioinformatics tools, miRDB (http://www.mirdb.org) and TargetScan (http://www.targetscan.org). The VEGFA 3′-UTR sequences containing the wild-type or mutant miR-206 binding sites were then constructed and inserted into the pGL6 luciferase reporter vector (Beyotime Institute of Biotechnology). HUVECs were cotransfected with the luciferase reporter vector plus miR-206 agomir or control using Lipofectamine 2000® (Thermo Fisher Scientific, Inc.). Following 48 h of transfection, the cells were harvested for luciferase activity detection following the instructions provided with the Dual-Luciferase Reporter Assay System (Promega Corporation).

### 2.12. Western Blot Analysis

Total protein from was extracted from the HUVECs and electrophoresed with 12% SDS-PAGE, followed by transfer onto PVDF membranes (Invitrogen; Thermo Fisher Scientific, Inc.). Subsequently, the membranes were blocked with 5% skim milk and then incubated with the following primary antibodies at 4°C overnight: VEGFA, p-AKT, p-ERK (1 : 500), AKT, ERK, CDK4, Cyclin D1, and cleaved caspase-3 (1 : 1,000). After washing with TBST three times, the membranes were incubated with specific secondary antibodies labeled with HRP for 1 h. Finally, the membranes were visualized using the ECL chemiluminescent substrate kit (Thermo Fisher Scientific, Inc.) and analyzed using ImageJ software (National Institutes of Health). The antibodies were all obtained from Cell Signaling Technology, Inc. *β*-Actin was used as the internal standard.

### 2.13. Detection of Oxidative Stress Indicators

The indicators of oxidative stress, including superoxide dismutase (SOD) activity, glutathione (GSH), and malondialdehyde (MDA) content, were examined. All assays were performed as per the manufacturer's protocols (Nanjing Jiancheng Bioengineering Institute).

### 2.14. Statistical Analysis

All experiments were repeated at least three times. The data were analyzed using GraphPad Prism software (GraphPad Software Inc.) and presented as the mean ± SD. The Student's *t*-test (two-tailed) and one-way ANOVA were applied to compare results between 2 groups or among multiple groups. *P* < 0.05 was considered to indicate a statistically significant difference.

## 3. Results

### 3.1. Profiling of Differentially Expressed miRNAs in Propranolol-Treated HUVECs

**Figure 1:**
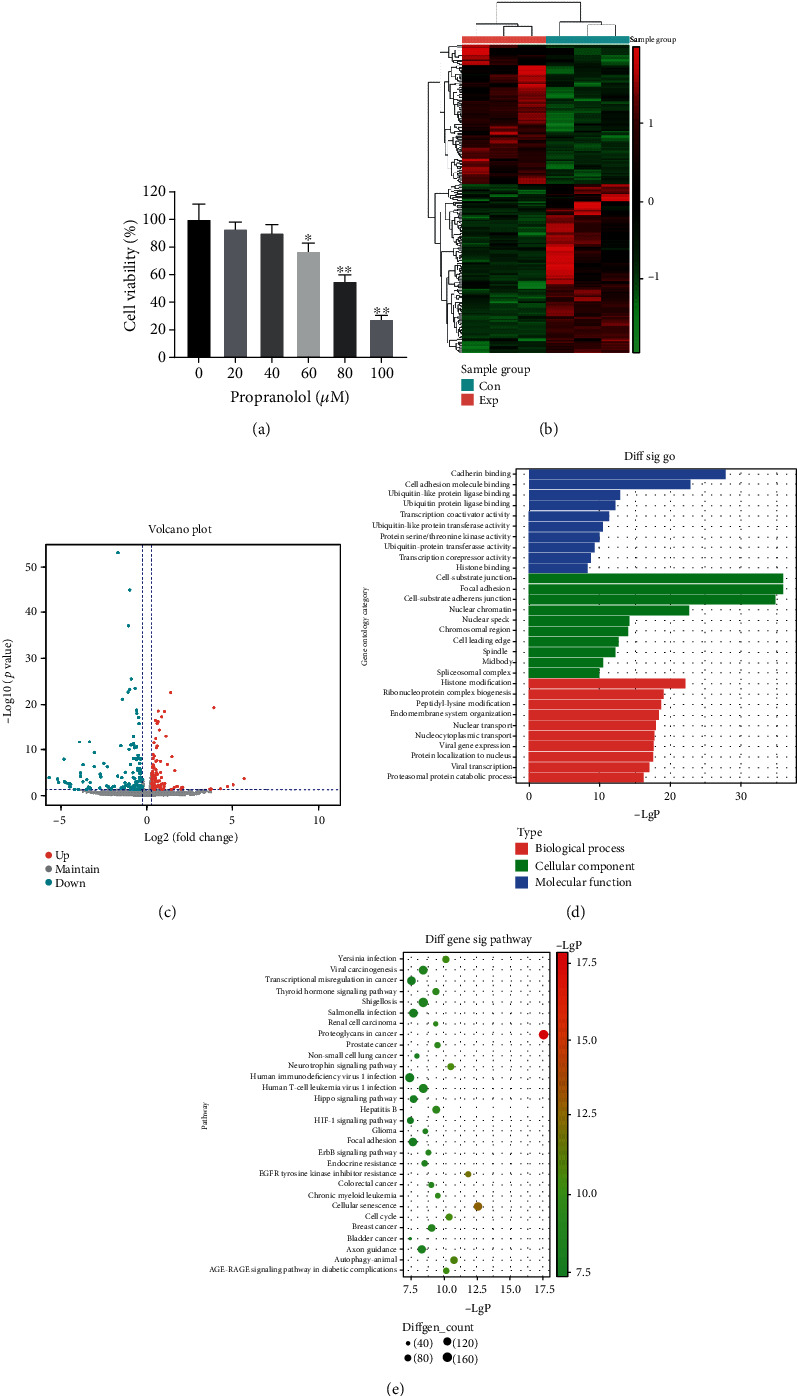
DEG analysis of HUVECs treated with propranolol. (a) Cell viability was determined by CCK-8 assay. *N* = 3, ^∗^*P* < 0.05, ^∗∗^*P* < 0.01, all comparing with the 0 *μ*M propranolol group. (b) Heat map showing the DEGs in cells treated with 80 *μ*M propranolol comparing with control cells. Red represents upregulated genes, green represents downregulated genes, and black represents unchanged genes, *N* = 3. (c) Volcano plot map showing the significantly upregulated (*P* value < 0.05, fold change > 1.2) and downregulated (*P* value < 0.05, fold change < 0.833) miRNAs. (d) GO enrichment analysis of protein targets of DEGs. (e) KEGG pathway analysis of protein targets of DEGs.

HUVECs were treated with serial concentrations of propranolol (0, 20, 40, 60, 80, or 100 *μ*M). The results of the CCK-8 assay revealed that propranolol treatment led to a concentration-dependent decrease in cell viability at 48 h (Figure 1(a)). Subsequently, miRNA-seq was performed on three independent groups of HUVECs treated with or without 80 *μ*M propranolol. As shown in the clustering analysis of the differentially expressed miRNAs (Figure 1(b)), 97 miRNAs were identified to be upregulated (*P* value < 0.05; fold change > 1.2), and 119 miRNAs were downregulated (*P* value < 0.05; fold change < 0.833). Additionally, these differentially expressed miRNAs were presented in a volcano plot (Figure 1(c)). GO and KEGG analyses were then performed to analyze the enrichment functions and pathways of the upregulated target genes. GO analysis revealed that the upregulated targets were mostly involved in “cadherin binding,” “cell substrate junction,” and “histone modification” (Figure 1(d)). KEGG analysis revealed that the upregulated targets were mostly focused in “Proteoglycans in cancer,” “Shigellosis,” “cellular senescence,” and “Cell cycle” (Figure 1(e)).

### 3.2. Propranolol Exerts Antiproliferative Effects on HUVECs through Demethylation of the miR-206 Promoter

**Figure 2:**
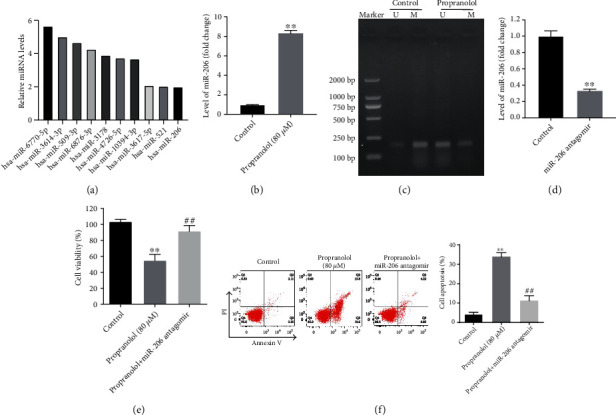
Propranolol inhibits survival of HUVECs through demethylation of pre-miR-206. (a) Top-ten upregulated miRNAs in propranolol-treated (80 *μ*M) HUVECs comparing with control cells. (b) HUVECs were treated with 80 *μ*M propranolol for 48 h, and the mRNA level of miR-206 was determined by RT-qPCR. (c) Methylation and unmethylation status of the pre-miR-206 gene were measured by MS-PCR. (d) The mRNA level of miR-206 in cells was determined by RT-qPCR after miR-206 antagomir transfection. (e) Cell viability was detected by CCK-8 kit. (f) Cell apoptosis was analyzed on flow cytometry after Annexin V/PI double staining. ^∗∗^*P* < 0.01, comparing with the control group; ^##^*P* < 0.01, comparing with the propranolol group; *n* = 3.

As shown by the results obtained for the differentially expressed miRNAs, miR-206 was one of the markedly upregulated miRNAs in the propranolol-treated HUVECs (Figure 2(a)). miR-206 has been demonstrated to play a key role in the vascular function of various cancer cells [20, 21]; thus, the association between miR-206 and propranolol in HUVECs was then investigated in the following experiments. The results of RT-qPCR further demonstrated that treatment with 80 *μ*M propranolol significantly upregulated the miR-206 level in the cells (Figure 2(b)). As is known, methylation in the CpG island can result in the downregulation of miRNAs [22]. Thus, methylation-specific PCR was performed to verify the methylation status of the miRNA-206 promoter. Compared with the control cells, propranolol treatment led to a decrease in the M promoter and an increase in the U promoter (Figure 2(c)). In order to further determine the role of miR-206 in propranolol-treated HUVECs, miR-206 antagomir was transfected into the cells. RT-qPCR confirmed that miR-206 antagomir notably decreased the expression of miR-206 in HUVECs (Figure 2(d)). Furthermore, propranolol significantly inhibited the growth of HUVECs by inducing apoptosis (Figures 2(e) and 2(f)), while this phenomenon was completely revered by miRNA-206 antagomir. Taken together, propranolol significantly inhibited the growth of HUVECs via demethylation of the miR-206 promoter.

### 3.3. Downregulation of miR-206 Reverses the Propranolol-Induced Inhibition of the Migration of HUVECs

**Figure 3:**
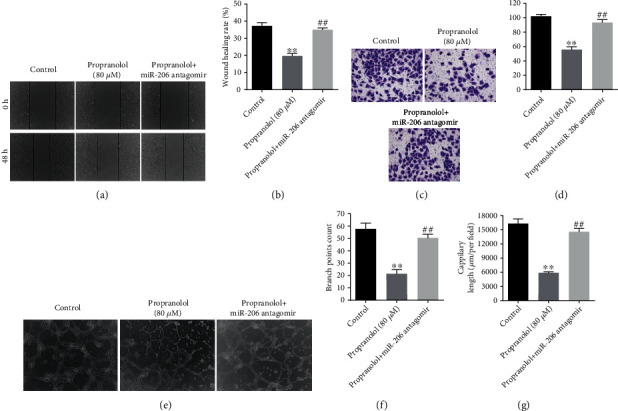
Downregulation of miR-206 reverses propranolol-induced inhibition of cell migration in HUVECs. HUVECs were divided into three groups: control, 80 *μ*M propranolol, 80 *μ*M propranolol + miR-206 antagomir. (a) and (b) Representative scratching area of cells and the calculated wound healing rate were indicated. (c) and (d) Migrated cells were stained with 0.2% crystal violet and counted at 3 random fields. (e) Representative images of tube formation were captured by microscopy at 24 h. (f) and (g) Branch points counts and capillary length were calculated at 3 random fields. ^∗∗^*P* < 0.01, comparing with the control group; ^##^*P* < 0.01, comparing with the propranolol group; *n* = 3.

A previous study suggested that propranolol can inhibit the migration of HUVECs [23]. In order to further explore the role of miR-206 in the propranolol-mediated inhibition of cell migration, Transwell, wound healing, and tube formation assays were conducted. As was expected, propranolol significantly inhibited the wound healing rate of HUVECs, while miR-206 antagomir notably reversed this effect (Figures 3(a) and 3(b)). Likewise, Transwell assay further confirmed that miR-206 antagomir reversed the propranolol-induced inhibition of cell migration (Figures 3(c) and 3(d)). In addition, the results of tube formation assay indicated that propranolol significantly decreased the number of branch points and the length of capillaries, whereas these phenomena were notably reversed by miR-206 antagomir. Taken together, the propranolol-induced inhibition of cell migration was reversed by miR-206 inhibition.

### 3.4. VEGFA Is a Potential Binding Target of miR-206

**Figure 4:**
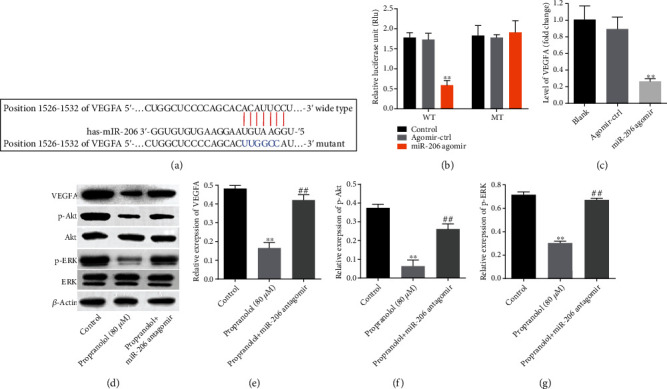
VEGFA is a target gene of miR-206. (a) Predicted binding sequences between miR-206 and VEGFA. (b) The relationship between miR-206 and VEGFA was verified by dual-luciferase reporter assay. ^∗∗^*P* < 0.01, comparing with the agomir control group; ^##^*P* < 0.01, comparing with the propranolol group; *n* = 3. (c) The mRNA level of VEGFA in HUVECs was detected with RT-qPCR after miR-206 antagomir transfection. (d)–(g) The protein levels of VEGFA, p-Akt, Akt, p-ERK, and ERK were determined by western blotting, and the relative expression of proteins was normalized with *β*-actin. ^∗∗^*P* < 0.01, comparing with the control group; ^##^*P* < 0.01, comparing with the propranolol group; *n* = 3.

To further investigate the role of miR-206 in propranolol-treated HUVECs, the potential target of miR-206 was searched using the miRDB and TargetScan databases. Both databases revealed that VEGFA may be a possible target gene of miR-206. The binding sequences between miR-206 and the 3′-UTR of VEGFA are presented in Figure 4(a). Furthermore, dual-luciferase reporter assay revealed that miR-206 agomir significantly reduced the luciferase activity of wild-type VEGFA, while it had no effect on the mutant one (Figure 4(b)). Moreover, miR-206 agomir notably suppressed the level of VEGFA in HUVECs (Figure 4(c)). Additionally, propranolol decreased the levels of VEGFA, p-AKT, and p-ERK in HUVECs, whereas these phenomena were significantly reversed by miR-206 antagomir (Figures 4(d)–4(g)). All these results illustrated propranolol suppressed the proliferation and migration of HUVECs through the regulation of the miR-206/VEGFA axis.

### 3.5. Downregulation of miR-206 Abolishes the Antioxidant Capacity of Propranolol in HUVECs

**Figure 5:**
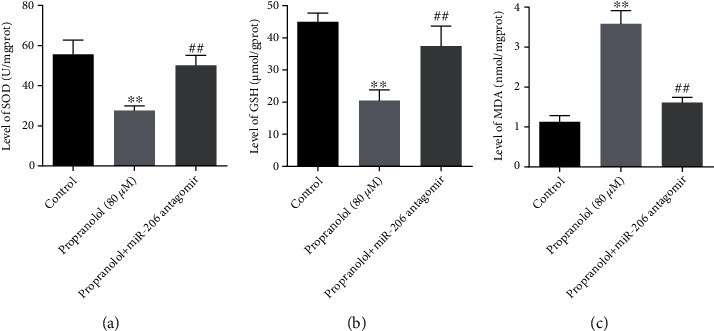
Downregulation of miR-206 abolishes the antioxidant capacity of propranolol in HUVECs. Cells were treated with propranolol or propranolol+miR-206 antagomir, the changes of (a) SOD activity, (b) GSH level, and (c) MDA level in HUVECs were measured, respectively. ^∗∗^*P* < 0.01, comparing with the control group; ^##^*P* < 0.01, comparing with the propranolol group; *n* = 3.

Since propranolol has been demonstrated to prevent angiogenesis via abolishing oxidative stress [24], indicators of oxidative stress were then detected. As shown in Figures 5(a) and 5(b), the activity of SOD and the contents of GSH were decreased following propranolol treatment; however, miR-206 antagomir reversed these effects. In addition, the level of MDA was notably increased by propranolol, and this effect was notably reversed by miR-206 inhibition. All these data suggested that propranolol exerted its antioxidant effects on HUVECs through the modulation of miR-206.

### 3.6. Downregulation of miR-206 Eliminates Propranolol-Induced Cell Cycle Arrest of HUVECs

**Figure 6:**
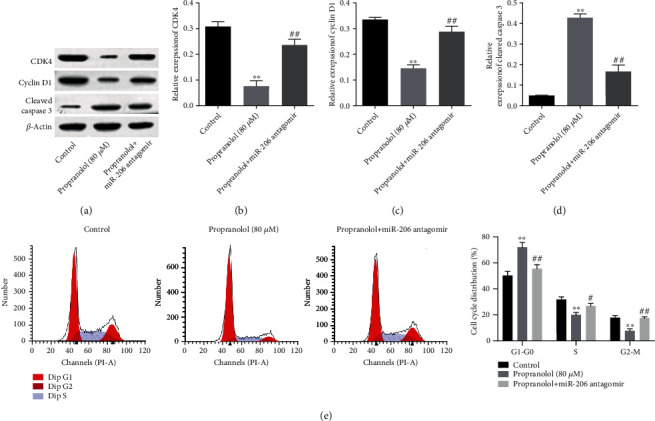
Downregulation of miR-206 eliminates propranolol-induced cell cycle arrest in HUVECs. Cells were treated with propranolol or propranolol+miR-206 antagomir. (a) The protein levels of CDK4, Cyclin D1, and cleaved caspase 3 were detected by western blotting. (b)–(d) The relative expression levels of CDK4, Cyclin D1, and cleaved caspase 3 were quantified by normalizing to *β*-actin. (e) Cell cycle distribution was analyzed by flow cytometry, and the result was quantified by flowjo software. ^∗∗^*P* < 0.01, comparing with the control group; ^##^*P* < 0.01, comparing with the propranolol group; *n* = 3.

As revealed by KEGG pathway analysis, the targets of the differentially expressed miRNAs exhibited a close association with “Cell cycle,” and previous reports revealed that miR-206 could inhibit the cancer cell growth via mediation of cell cycle proteins [25, 26]. Thus, cell cycle behavior was then detected in the following experiment. As was expected, the expression levels of CDK4 and cyclin D1 were decreased following propranolol treatment, while miR-206 antagomir significantly reverse these effects (Figures 6(a) and 6(b)). On the other hand, the propranolol-induced upregulation of cleaved caspase-3 expression was notably reversed by miR-206 antagomir (Figure 6(c)). Additionally, cell cycle analysis revealed that G1 arrest induced by propranolol in the HUVECs was notably eliminated by miR-206 inhibition. Taken together, these data suggested that the propranolol-induced cell cycle arrest of HUVECs was eliminated by miR-206 inhibition.

## 4. Discussion

For decades, it has been known that miRNAs function as key regulators of vascular diseases. In 2016, Strub et al. [27], for the first time, found that miRNA-C19MC was a biomarker of IH. The level of circulating C19MC was associated with the IH tumor size and the response to propranolol treatment [27]. Thereafter, other studies found that miRNAs regulated the process of IH through various mechanisms. For example, Li et al. [28] found that propranolol treatment led to the downregulation of miR-382 via the PTEN/AKT/mTOR pathway in XPTS-1 cells. Mong et al. also revealed that the LIN28B/Let-7 signaling axis was involved in the propranolol-induced involution of IH [29]. As is known, due to its ability to decrease DNA synthesis [30], miR-206 has been shown to reduce angiogenesis [31], thus, suggesting that it may effectively inhibit cancer development [20, 32]. Recent studies have demonstrated that miR-206 can inhibit the proliferation, invasion, and migration of a number of cancer cells [20, 33, 34]. Similar to these studies, the present study found that miR-206 was involved in the antiangiogenic effects of propranolol on HUVECs.

Tumor angiogenesis involves endothelial cell proliferation and migration, which are activated through the VEGF/VEGFR pathway [35, 36]. As aforementioned, miR-206 plays an antiangiogenic role in different tumor types. There is evidence to indicate that miR-206 targets the VEGFA/CCL2 signaling pathway to inhibit tumor progression [37]. Another study found miR-206 suppressed the Met/ERK/Elk-1/HIF-1*α*/VEGF-A pathway in CCL19-mediated colorectal cancer cells [21]. In the present study, it was found that VEGFA was a target of miR-206, and that miR-206 antagomir reversed the propranolol-induced downregulation of VEGFA in HUVECs. Another study also demonstrated that propranolol reduced the expression of VEGF and VEGF-A via the downregulation of miR-4295 in HUVECs [23]. Thus, there may be other signaling pathways participating in this process, which warrants further investigation into this matter in the future.

In the present study, an important finding was that the “Cell cycle” was involved in the upregulated miRNAs, as revealed using KEGG analysis. The role of the cyclin D/CDK axis in angiogenesis has already been documented [38]. Additionally, researchers have found that miRNAs affect the progression of the cell cycle by targeting the CDK1 and 4/6 genes [39] or E2F transcription factor 8 [40]. Although the cell cycle-related proteins were not found to be the direct target of miR-206 in the present study, cell cycle arrest was observed in the propranolol-treated HUVECs.

Another interesting finding of the present study was that propranolol treatment decreased the methylation level of pre-miR-206. DNA hypermethylation in the promoter is the most frequent mechanism leading to downregulated miRNAs [41]. Previously, researchers have found aberrant methylation status of miRNAs in various types of cancer [42, 43], and methylated miRNAs have been shown to be involved in cancer cell proliferation [44, 45]. This finding may lead to the better understanding of the mechanisms through which propranolol regulates the progression of IH.

## 5. Conclusions

In conclusion, the findings of the present study demonstrated that propranolol may suppress the proliferation and migration of HUVECs through the regulation of the miR-206/VEGFA axis. These findings partly explain the mechanisms through which propranolol regulates the progression of IH and may aid in the development of novel therapies and drugs in the future.

## Data Availability

The datasets used and/or analyzed during the current study are available from the corresponding author on reasonable request.
